# Antenatal care and determinants of obstetric danger signs awareness of immediate postpartum women at Buea Regional Hospital, Cameroon

**DOI:** 10.11604/pamj.2021.38.247.20977

**Published:** 2021-03-08

**Authors:** Agbor Nathan Emeh, Atem Njabnjem Atem, Atongno Ashu Humphrey, Tambetakaw Njang Gilbert, Fongang Che Landis

**Affiliations:** 1 Department of Public Health and Hygiene, Faculty of Health Sciences, University of Buea, Buea, Cameroon,; 2 Alpha Higher Institute of Douala, Douala, Cameroon,; 3 Ministry of Public Health, Yaoundé, Cameroon,; 4 Department of Health Sciences, Cameroon Christian University, Cameroon

**Keywords:** Obstetric danger signs, determinants, postpartum, antenatal care

## Abstract

**Introduction:**

a significant proportion of pregnancy related deaths result from delay in decision to seek care and this often stems from failure to identify obstetric danger signs earlier. Early identification of these danger signs will therefore reduce maternal mortality. However, studies on obstetric danger signs awareness are lacking in Cameroon. The objective of this study was to assess the determinants of obstetric danger signs awareness of women at immediate postpartum period. This will inform ANC providers´ practice.

**Methods:**

between June and September 2019, women who delivered at the Buea Regional Hospital were interviewed within 24 hours following their delivery using a researcher-administered questionnaire that covered socio-demographic and obstetric variables. Data were entered into EpiData and analysis done using SPSS 16 and OpenEpi. Statistical significance was set at p-value < 0.05.

**Results:**

of the 532 participants, majority (230/532: 43.2%) were those aged 26-35; danger signs awareness rate was 73.3%. There was a statistically significant relation between age and awareness of obstetric danger signs which showed that older women were more aware than their younger counterparts (p=0.00). Other statistically significant determinants of danger sign awareness included occupation, level of education, parity, trimester of onset of antenatal visits and the number of visits before delivery (p<0.05). Multiparity (370/490: 75.5%) and grand multiparity (14/22: 63.6%) were more likely to be aware of obstetric danger signs than primiparous women (6/20: 30%). Similarly, those who started antenatal visits earlier (first or second trimester) and those who attended more visits were more likely to be aware of obstetric danger signs than their counterparts who started later or had lesser antenatal visits before delivery. The most reported danger signs were severe vaginal bleed (71.4%), fever (62.0%) and reduced fetal movement.

**Conclusion:**

conclusively, more focus should be placed on the sensitisation about obstetric danger signs when in contact with primiparous and younger parturient during ANC visits.

## Introduction

Maternal mortality remains a major public health problem particularly in the developing countries [1]. About 295000 women died in 2017 during and following pregnancy and childbirth with over 94% of those deaths occurring in less developed countries [2] where maternal mortality ratio (MMR) is over 15 times higher than in the developed regions [3]. Although sub-Sahara as a region has achieved nearly 40% reduction in MMR since 2000, the region and Southern Asia alone accounted for approximately 86% of the estimated global maternal deaths in 2017 most of which are preventable [4]. Each pregnancy is associated with an inherent risk of sudden, unpredictable complications that could end up with the death or injury of the woman or her infant [5].

World bank sources indicate that Cameroon has witnessed a rise in maternal mortality ratio (MMR) from 728 in 1990 to 753 in 1996 followed by a relatively steady ratio of 751 between 1997 to 2001; then steady drop from 751 in 2001 to 596 in 2015 making Cameroon rank number 15 country with high MMR in the world [6]. Although there has been an apparent drop in MMR in Cameroon, the country is far from reaching its recommended MDG targets [7]. Most of the maternal deaths are preventable and have been attributed to three delays: delay in the decision to seek care, delay in reaching the place of care, and delay in receiving appropriate care [8]. Direct obstetric causes account for over 80% of maternal deaths worldwide, including hemorrhage, infection, obstructed and prolonged labor, unsafe abortion and hypertensive disorders of pregnancy [9]. When the awareness of obstetric danger signs is poor, women tend to delay in seeking obstetric care and this can result in increased maternal mortality and morbidity [10]. Women´s knowledge on these danger signs is still very low in sub-Saharan Africa [10-12]. Health education on obstetric danger signs has been used to increase utilisation of skilled obstetric care when complications are anticipated [13]. Some developing countries are now making efforts to increase awareness of obstetric danger signs through implementation of focused antenatal care (FANC) which provide free counselling on these danger signs to all pregnant women attending ANC [14, 15]. Even though the proportion of pregnant women attending ANC at least once is often high, at times reaching 98% [16], very few of them admit haven been informed on obstetric danger signs [10]. Despite efforts made to increase awareness on these danger signs, studies still show that very few pregnant women in LDCs receive information on these danger signs during ANC [17].

Factors that have been associated with level of knowledge of obstetrical danger signs include maternal age [18], level of education [12, 19], employment status [20], number of ANC visits [21] and parity status [20]. The aim of this study was to assess the level of awareness on obstetric danger signs among pregnant women; assess the attitude of pregnant women towards obstetric danger signs and; evaluate the relationship between socio-demographic determinants and awareness on obstetric danger signs. This information is vital because it is estimated that it will help ANC providers to orientate their health education programs on pregnant women towards obstetric danger signs during their routine ANC visits.

## Methods

**Study design**: this study was an analytic cross-sectional study.

**Study area**: Buea is found in Fako Division in the South West Region of Cameroon. It covers a total surface area of 870 square km. It has an equatorial climate, and temperatures range between 20-28°C. The town experiences two major seasons; a rainy season that begins in March and ends in October, and a dry season that begins in November and ends in February. Annual rainfall varies from 3000 to 5000 mm.

**Study setting**: the Buea Regional Hospital is a secondary care centre located in the Buea Health District which was created in 1995. The hospital acts as the referral centre for the Health District which is the 3^rd^ most populated in the South West Region with an estimated population of 173,526 in 2018. During the time of the study, routine antenatal care visits were carried out by midwives and state registered or assistant nurses who also carried out all deliveries. Cases that required more monitoring were followed-up by Obstetricians or General Practitioners who also carried out caesarean deliveries.

**Study duration**: this study was conducted from June to September, 2019.

**Sample size**: the sample size for the study was calculated using the StatCalc of Epi Info software, version 7.2.2.16 using the following parameters: Buea Health District population size=173,526; Expected frequency=50%; acceptable margin of error=5%; design effect=1; 95% confidence level. This gave us a minimum sample size of 383. However, we sampled 532 women who had delivered in the Buea Regional Hospital and were at their immediate postpartum period during the study period.

**Inclusion and exclusion criteria**: those included in the study were women who were at their immediate postpartum period (within 24 hours following delivery) and had attended ANC in a health facility within the Buea Health District and delivered at the Buea Regional Hospital. Each woman was required to give her consent before she could be included in the study. Those who refused to give consent or were mentally retarded or did not meet the above inclusion criteria were excluded from the study.

**Sampling and data collection**: during the study period (June to September, 2019), data were collected from Monday to Sunday each week. Trained data collectors met all participants, who were immediate postpartum women at the Buea Regional Hospital´s maternity wards, where they presented the purpose of the study and data were obtained from those who provided their consent using pretested researcher-administered questionnaire. Each participant was interviewed once throughout the study period.

**Tool**: the questionnaire was designed by two reproductive health experts and was pretested on 20 pregnant women who attended antenatal care in the same hospital. However, the results of the pretested questionnaires were not included in the final data analysis and participants who were used for the pre-test did not take part in the study. The questionnaire was design to cover socio-demographic data, including age, marital status, educational level, etc. Obstetrical data collected included gravida status and antenatal care visits. The questionnaire also captured the level of awareness and the parturient experience of obstetric danger signs.

**Data management**: data were entered and cleaned using EpiData 3.1, then exported and analysed in SPSS version 16 and OpenEpi. Statistical significance was considered at p<0.05. Categorical variables were compared using Chi-square for trend test.

**Ethical considerations**: ethical clearance was obtained from the Institutional Ethics Committee for Research on Human Health of the University of Douala (Nº 1987 IEC-UDo/06/2019/M). Administrative authorisation was also obtained from the Buea Regional Hospital. Each participant either gave consent, signed or verbal before they could be included in the study. Refusal to participate in the study did not affect patient care.

### Operational definitions

**Obstetric danger signs**: obstetric danger signs are unexpected obstetric signs that can lead to maternal health complications [10]. These danger signs are mainly classified into three categories. Major danger signs during pregnancy include: severe vaginal bleeding, swollen hands/face, and blurred vision. Major danger signs during labor and childbirth include severe vaginal bleeding, prolonged labor (>12 h), convulsions, and retained placenta. Major danger signs during the postpartum period include severe vaginal bleeding, foul-smelling vaginal discharge, and fever [22]. However, we cannot limit ourselves to these few signs since there exist several other such obstetric signs that may signal impending danger to the mother and child, including decreased fetal movements, anemia/palor and high blood pressure.

**Antenatal care**: care provided by skilled health care professionals to pregnant women in order to ensure the best health outcomes for both the mother and baby during pregnancy [23].

**Postpartum period**: also known as the puerperium refers to the time after delivery when maternal physiological changes related to pregnancy return to the non-pregnant state. It is well accepted that this period begins upon delivery of the new-born but when it ends has remained controversial as some authors consider six to eight weeks [24] and even up to 6 months [25]. Three postpartum phases with distinct care and needs are: initial or acute period (6-12hours), sub-acute postpartum period (2-6 weeks) and delayed postpartum (up to 6 months) [25, 26]. Our study was limited to participants of the immediate postpartum period (6-12 hours after delivery).

**Limitation**: being a hospital-based study means the danger signs´ awareness rate does not represent the information of those women who delivered at home or at traditional birth attenders´ delivery site (and consequently are those who do not attend ANC). Thus the level of awareness is expected to be higher than in community-based study. The prevalence of severe bleeding in this study may represent a lower value since participants were immediate postpartum women and consequently will miss those who may suffer severe bleeding in their late postpartum period. In this study, we did not assess awareness and experience of certain danger signs such as foul-smelling vaginal discharge since they will only occur in the late postpartum period.

## Results

**General characteristics**: during our study period, we interviewed 540 postpartum women but finally retained 532 for analysis due to withdrawal of 8 participants for insufficient information in their study questionnaires. The participants had an age range of 15-51 (Mean; 30.27). Majority were women aged 26-35 years old [230/532; 43.2%], married [258/532; 48.5%], low income [298/532; 56.0%] and multiparous [490/532; 92.1%] (Table 1).

**Table 1:** general characteristics and determinants of obstetric danger signs awareness

Variables	Participants N=532; n1; (n1/N %)	Awareness of obstetric danger signs; n2 (n2/n1 %)	OR [95% CI]	p-value
**Age range in years**				
15-25years	156 (29.3)	90 (57.7)	1	<0.001*
26-35	230 (43.2)	184 (80.0)	2.93	
36-45	138 (25.9)	108 (78.3)	2.64	
Above 46	8 (1.5)	8 (100.0)	-	
**Marital status**				
Single	200 (37.6)	130 (65.0%)	1	0.15
Married	258 (48.5)	206 (79.8)	2.13	
Divorced	6 (1.1)	6 (100.0)	-	
Widowed	68 (12.8)	48 (70.6)	1.29	
**Occupation**				
Traders	170 (32.0)	96 (56.5)	1	<0.001*
House wife	158 (29.7)	140 (88.6)	5.99	
Employed by the Government	98 (18.4)	78 (79.6)	3.01	
**Employed by a private institution**	106 (19.9)	76 (71.7)	1.95	
**Level of education**				
Primary	96 (18.1)	44 (45.8)	1	<0.001*
Secondary	186 (35)	138 (74.2)	3.40	
Vocational	86 (16.2)	64 (74.4)	3.44	
University	164 (30.8)	144 (87.8)	8.51	
**Monthly income (FCFA)**				
<50000	298 (56.0)	208 (69.8)	1	0.18
50000-100000	194 (36.5)	154 (79.4)	1.67	
>100000	40 (7.5)	28 (70.0)	1.01	
**Parity**				
Primiparous	20 (3.8)	6 (30.0)	1	0.04*
Multipara	490 (92.1)	370 (75.5)	7.19	
Grand Multipara	22 (4.1)	14 (63.6)	4.08	
**Trimester of onset of ANC visit**				
1st trimester	348 (65.4%)	274 (78.7)	1	<0.001*
2nd trimester	180 (33.8%)	116 (64.4)	0.49	
3rd trimester	4 (0.8%)	0 (0)	0.00	
**Number of ANC visits attended before delivery**				
0 or 1	57 (10.7)	30 (52.6)	1	<0.001*
2	176 (33.1)	120 (68.2)	1.93	
3	164 (30.8)	126 (76.8)	2.98	
4	86 (16.2)	66 (76.7)	2.97	
>4	49 (9.2)	48 (98.0)	43.2	

* = Statistically significant

**Figure 1:**
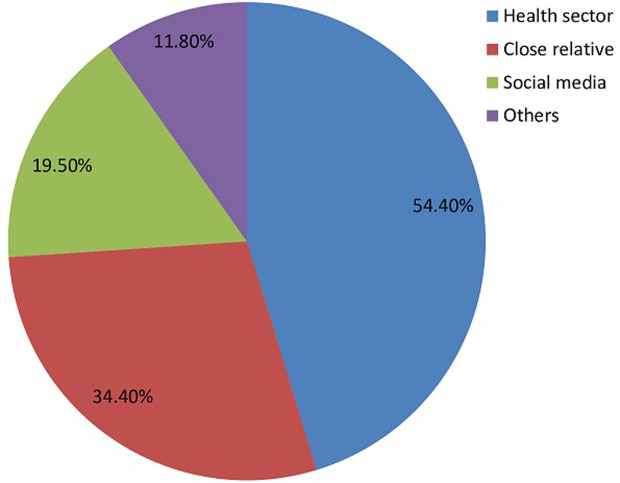
main sources of information on obstetric danger signs

**Awareness of danger signs of pregnancy**: three hundred and ninety [390/532; 73.3%] women were aware of obstetric danger signs while more than half [344/532; 64.7%] attested to have received some form of education on obstetric danger signs at some point in time during their antenatal visits. Among the 390 women who were aware of obstetric danger signs, 54.4% [212/390] identified the health sector as their main sources of information on obstetric danger signs, followed by relatives and friends [134/390; 34.4%] (Figure 1).

**Figure 2:**
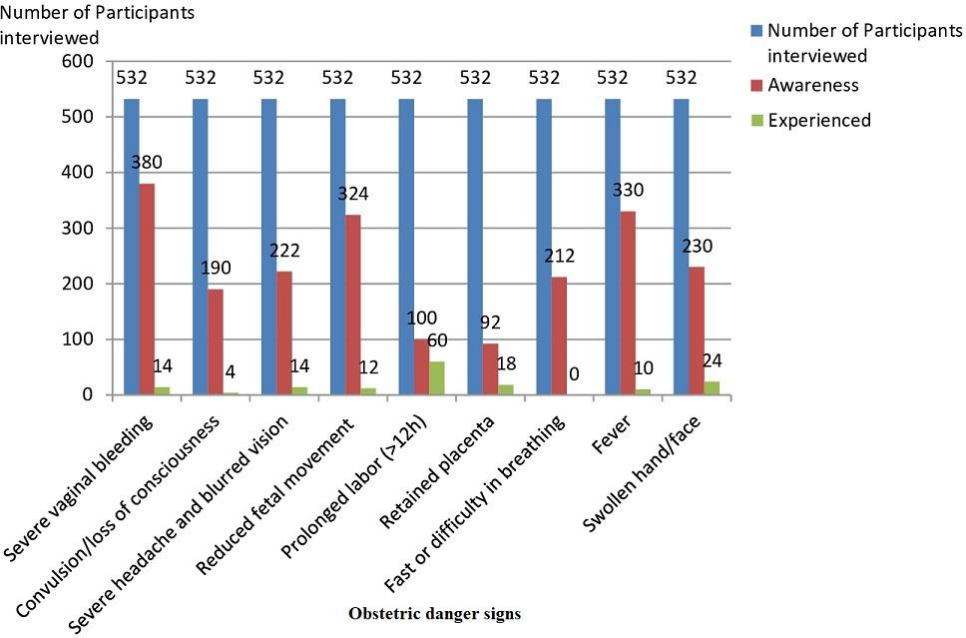
level of awareness of obstetric danger signs and prevalence among immediate postpartum women

The most known obstetric danger signs were severe vaginal bleeding [380/532; 71.4%], fever [330/532; 62.0%], reduced fetal movements [324/532; 60.9%] and edema [230/532; 43.2]. Most common obstetric danger signs encountered during pregnancy by participants were prolong labor [60/532; 11.3%], edema [24/532; 4.5%], retained placenta [18/532; 3.4%], severe bleeding [14/532; 2.6%] and severe headache/blurred vision [14/532; 2.6%] (Table 2, Figure 2).

**Table 2:** awareness and experience of obstetric danger signs (N = 532)

Danger signs	Awareness (%) (p<0.001)	Experienced (%) (p<0.001)
Severe vaginal bleeding	380 (71.4)	14 (2.6)
Convulsion/loss of consciousness	190 (35.7)	4 (0.8)
Severe headache and blurred vision	222 (41.7)	14 (2.6)
Reduced fetal movement	324 (60.9)	12 (2.3)
Prolonged labor (>12h)	100 (18.8)	60 (11.3)
Retained placenta	92 (17.3)	18 (3.4)
Fast or difficulty in breathing	212 (39.9)	0 (0)
Fever	330 (62.0)	10 (1.9)
Swelling of fingers, face and legs (odema)	230 (43.2)	24 (4.5)

**Determinants of awareness obstetric danger signs**: Table 1 shows determinants of awareness of obstetric danger signs. Statistically significant determinants of obstetric danger sign awareness included the age of the woman, occupation, level of education, parity, the trimester at the woman began her antenatal care (ANC) and the number of ANC visits the woman attended with p-values of 0.00, 0.00, 0.00, 0.04, 0.00, 0.00 respectively. Women of age above 46 years were more aware [8/8; 100%] than those of younger ages (<46year). Higher level of educations were associated with higher levels of awareness of obstetric danger signs compared to those with lower levels of education with univariate OR for primary, secondary, vocational and university levels being 1, 3.40, 3.44, and 8.51 respectively. Those who started ANC at their first trimester were more aware [274/384; 78.7%] than those who started at their second [166/180; 64.4%] and third trimester [0/4; 0%]. Similarly, those who attended more ANC visits were more likely to be aware of obstetric dangers signs than those who attended less with OR of 1, 1.93, 2.98, 2.97, and 43.2 for those who attended once, twice, trice, 4 visits and 5 and above visits respectively.

## Discussion

Pregnancy related deaths in Cameroon remain unacceptably high [27], yet studies in the country addressing awareness of important pregnancy concepts such as obstetric danger signs are non-existent. A number of studies assessing obstetric danger signs awareness have been conducted in other parts of the globe, most of which are community-based studies. The rationale for this hospital-based study was to capture fresh memories of obstetric danger signs awareness by interviewing participants at their immediate postpartum period.

In our study, 73.3% of participants were aware of obstetric danger signs. This value is higher when compared to the level of awareness reported in other studies [5, 12]. This difference in the level of awareness may be as a result of difference in the study design. It will be reasonable to expect higher levels of reported awareness in immediate postpartum participants as in the case of our study when compared to studies whose participants are later postpartum women and those who have delivered long before their interview due to memory bias as in the case of the other studies [5]. Also, higher level of education has been reported as the most important predictive factor for increased awareness [5, 12]. Buea Health District harbours several higher institutions of learning, including the University of Buea. It will be expected therefore that there should be more educated women in this area, thus contributing to the high level of awareness of obstetric danger signs. Those who attended University level were over 8 times more likely to be aware than primary and lower levels of education. Kumbani and McLnerney insisted that better education is associated with enlightenment and awareness of different health conditions although exposure to information is crucial [12, 28]. This might be a contributing mechanism through which higher educational level has been associated with lower maternal mortality [29]. There was no statistically significant difference between level of awareness of obstetric danger signs and income. This differs from the observation made by Yinager and collaborators where he established that higher monthly income was associated with higher awareness and justified the notion that money is needed to utilize health related services at any time the woman want [5], including antenatal visits.

The most recognized danger signs reported in our study included severe vaginal bleeding, fever, reduced fetal movement, edema and severe headache/blurred vision. Although in our study, we did not make any distinction of obstetric danger signs at the usual three different periods (during pregnancy, delivery and after delivery), vaginal bleeding is the most aware obstetric danger signs reported by other studies [10, 12, 30]. Awareness of vaginal bleeding as the most recognised obstetric danger sign may probably be because it is the most visible sign and the most common cause of maternal death [31]. Few participants recognized prolonged labour as an obstetric danger sign. Unlike a study in Pakistan that reported a slightly higher awareness of prolonged labor as a danger sign [30], studies in Gambia [32], Malawi [28] and Tanzania [12] have either failed to recognize prolonged labour as an obstetric danger sign or have reported low awareness of prolonged labour as an obstetric danger signs. The reason for these differences remains unknown. However, we presume that cultural differences in these different settings may be implicated in that differences in the perception of what prolonged labor is defined to be may preclude its recognition as an obstetric danger sign.

Predictive factors of obstetric danger sign awareness assessed in our study included age of the participants, their marital status, occupation, level of education, monthly income, parity, trimester at which parturient began ANC and the number of antenatal care visits. The most predictive factors for obstetric danger signs awareness were the level of education, trimester at which ANC was started and number of antenatal visits attended before delivery, such that the awareness was higher among those with highest level of education and lowest among those with lowest level of education. Similarly, those who attended more ANC visits before delivery were more likely to be aware of obstetric danger signs and lastly, parturient who began their ANC visits earlier (during first and second trimester) were very much likely to be aware of danger signs than those who started later. Pembe *et al*. had made a similar observation in their study in Tanzania [12]. This could be expected in that starting ANC early will increase chances of having more visits before delivery thereby increasing chances of being educated on obstetric danger signs. Our study also showed a high-level attendance of at least one ANC visit (502/532; 94.4%) among the women who delivered during our study period. It is important to note that all health facilities within the Buea Health District incorporate routine ANC health education including information on obstetric danger signs during their antenatal care sessions in accordance with the focus antenatal care (FANC) model adopted by the Cameroon Ministry of Public Health [33]. Therefore, the level of awareness of danger signs is likely to be relatively higher among the women who attend ANC visits in our study area compared to other studies [5, 12]. However, a recent Cochrane review has failed to prove high quality evidence for the benefit of antenatal education for childbirth [34].

Unlike a study in Ethiopia where age had no effect on the knowledge of obstetric danger sign [5], we noticed a statistically significant relationship between maternal age and level of awareness of obstetric danger signs such that the oldest women were more aware of obstetric danger signs. Similarly, multiparitous and grand multiparous women were more likely to be aware of obstetric danger signs than primiparous women. It is likely that older and multiparous women may have had some past experiences of obstetric danger signs in past pregnancies which may itself act as their own source of information and awareness. Therefore, younger women and those on their first pregnancy will require more counselling and health education on obstetric danger signs taking into consideration their inexperience in these danger signs [12].

## Conclusion

In summary, we noticed that age of the parturient, level of education, parity, trimester at which the parturient began antenatal visit and the number of antenatal visit attended by the parturient before delivery were the major determinants of awareness of obstetric danger signs. This is reasonable in that those who have lived longer (older parturient) or have had more deliveries before will have more chances of haven been informed by their past experiences thus more aware of obstetric danger signs. Similarly, starting antenatal visits earlier and having more ANC visits before delivery will increase chances that the parturient are educated on obstetric danger signs at some point in time before delivery. These findings therefore justifies the necessity for pregnant women to start antenatal care visits early and also to make effort to attend as many visits as required to ensure a favourable pregnancy outcome.

### What is known about this topic

- Delayed identification of obstetric danger signs is a risk factor for maternal dead;
- Most reported obstetric danger signs are vaginal bleeding and fever;
- Maternal age, level of education, employment status, number of ANC visits and parity status are associated to knowledge of obstetric danger signs.

### What this study adds

- Starting antenatal care early increases the chance that a parturient becomes aware of obstetric danger signs;
- Although further studies are needed to confirm this, multigravidity and older parturient are more likely to be aware of obstetric danger signs probably due to their past experiences.
